# Computational Simulations in the Cardiovascular System

**DOI:** 10.1155/2014/421061

**Published:** 2014-04-07

**Authors:** Aike Qiao, Hai-Chao Han, Makoto Ohta, Yi Qian

**Affiliations:** ^1^College of Life Science and Bioengineering, Beijing University of Technology, Pingleyuan 100, Chaoyang District, Beijing 100124, China; ^2^Department of Mechanical Engineering, The University of Texas at San Antonio, 1 UTSA Circle San Antonio, TX 78249, USA; ^3^Intelligent Fluid Systems Division Institute of Fluid Science, Tohoku University, 2-1-1 Katahira, Aoba-ku, Sendai, Miyagi 980-8577, Japan; ^4^Australian School of Advanced Medicine, Macquarie University, 2 Technology Place, NSW 2109, Australia

Cardiovascular diseases are the leading cause of mortality worldwide. Understanding the mechanism and developing techniques for diagnosis, treatment, and prevention remain as challenging issues. Biomechanics research plays an important role in all these aspects. Computer-based simulations of dynamic processes in the cardiovascular system are emerging as a powerful tool in delineating the biomechanical mechanisms and developing new treatment technologies for cardiovascular diseases. Although there have been extensive studies in these areas, many issues remain unsolved. The articles collected in this special issue presented new advances on the modeling and simulations of processes involving stenosis, clot, plaque, aneurysm, left ventricle syndrome, and so forth. These results reveal significant insights into the prediction, evaluation, and treatment of cardiovascular diseases. We hope that this special issue will also advance research in medical devices and surgical planning through computational simulations.

*Aike Qiao* *Aike Qiao* *Hai-Chao Han* *Hai-Chao Han* *Makoto Ohta* *Makoto Ohta* *Yi Qian* *Yi Qian*

